# Inhaled nitric oxide for critically ill Covid-19 patients: a prospective study

**DOI:** 10.1186/s13054-020-03371-x

**Published:** 2020-11-12

**Authors:** Osama Abou-Arab, Pierre Huette, Fanny Debouvries, Hervé Dupont, Vincent Jounieaux, Yazine Mahjoub

**Affiliations:** 1 Department of Anaesthesiology and Critical Care Medicine, Amiens Picardie University Hospital, 1 rue du Professeur Christian Cabrol, 80054 Amiens, France; 2 Respiratory Department, Amiens Picardie University Hospital, 80054 Amiens, France

Dear editor,

The role of inhaled nitric oxide (iNO) in the management of severe hypoxia due to coronavirus disease 2019 (Covid-19) is a subject of debate. Despite the lack of clinical data, the surviving sepsis campaign recommended the use of iNO as a rescue therapy in such patients with persistent hypoxemia and, at the same time, reminded that this treatment must be tapered off in the absence of rapid improvement [1].

The aim of the present study is to record the effect of iNO administration in COVID-19 patients with severe pneumonia.

We conducted a single-center prospective study at Amiens Hospital University (France), (ancillary study of a prospective COVID-19 critically patient database registered on ClinicalTrials.gov: NCT04354558 and declared to the CNIL number: PI2020_843_0026).

The population study was conducted on adults admitted in our intensive care unit for a COVID-19 severe pneumonia defined according to the WHO case definition [2]. All patients underwent a chest CT scan before iNO administration.

We administered 10 ppm of iNO (Kinox, Air Liquid Healthcare, Canada) through the inspiratory limb of the ventilator tubing when PaO_2_/FiO_2_ ratio was under 150 according to our local protocol management. Response to iNO was defined as an increase in PaO_2_/FiO_2_ over 20% during over 30 min following its administration. In the absence of response to iNO administration, patients received one session of prone positioning. The following respiratory parameters were collected at baseline and after 15 to 30 min of iNO administration: positive end expiratory pressure (PEEP), respiratory lung compliance (RS compliance), driving pressure, fraction in inspired oxygen (FiO_2_), PaO_2_, PaCO_2_ and the echocardiographic presence of an acute *cor pulmonale* (ACP).

Data were presented as median [interquartile range] or as number (percentage). Responders group and non-responders group were compared using Wilcoxon–Mann–Whitney, chi-2 or Fischer exact test, as appropriate. Statistical tests were performed using SPSS software version 24. A *P* value under 0.05 was considered as significant.

From 1st of March to 31st of May 2020, 34 of 80 patients with COVID-19 severe pneumonia received iNO. Twenty-two of 34 patients (65%) were responders and twelve were non-responders (35%). After iNO administration, PEEP, RS compliance and driving pressure remained un1

changed both in responders and in non-responders. At baseline, PaO_2_/FiO_2_ was significantly lower in the responders group in comparison with the non-responders group (respectively, 70 [63–100] vs 134 [83–173]; *P* < 0.0001) and was similar between groups after iNO administration (*P* = 0.068). PaCO_2_ levels were comparable between groups at baseline and after iNO administration. Prone positioning was not performed in the responders group.

We found a response rate of 65% to iNO administration. Our results differ from two recent reports on iNO use in COVID-19 in which the authors concluded in the absence of effectiveness of iNO [3, 4]. However, Tavazzi et al. found a positive effect in patients with ACP suggesting an effect of iNO on pulmonary circulation. Our results do not confirm this finding regarding the similar rate of ACP in the two groups. Among mechanisms of hypoxemia in COVID-19 patient, the presence of an intra-pulmonary shunt has been suggested [5]. In such hypothesis, the administration of iNO might worsen the shunt related to the pulmonary vasodilatation and might partially explain the decrease in PaO_2_/FiO_2_ in non-responders (Table 1). Regarding the CT scan features, we did not find any difference between groups, and thus, the absence of response to iNO could not be attributed to an increase “perfusion” of extensive ground glass opacities in non-responders.

**Table 1:** Data comparisons for responders and non-responders at baseline and after 15 to 30 min of nitric oxide (iNO) inhalation

Variables	Non-responder (*n* = 12)	Responder (*n* = 22)	*P* value
PaCO_2_ (mmHg)			
Baseline	49 [36–56]	48 [42–60]	0.363
After iNO	47 [42–60]	47 [38–52]	0.444
*P* value before/after iNO	*0.581*	*0.067*	
PaO_2_ (mmHg)			
Baseline	134 [80–160]	65 [58–86]	< 0.0001
After iNO	72 [68–108]	92 [73–131]	0.110
*P* value before/after iNO	< *0.009*	< *0.0001*	
PaO_2_/FiO_2_			
Baseline	134 [83–173]	70 [63–100]	< 0.0001
After iNO	125 [92–144]	144 [107–175]	0.068
*P* value before/after iNO	*0.005*	< *0.0001*	
FiO_2_			
Baseline	0.8 [0.7–0.9]	0.95 [0.7–1.0]	0.168
After iNO	0.75 [0.65–0.90]	0.70 [0.6–0.8]	0.557
*P* value before/after iNO	*0.399*	*0.002*	
PEEP (cmH_2_0)			
Baseline	12 [10–12]	12 [9–15]	0.790
After iNO	12 [10–13]	13 [9–15]	0.486
*P* value before/after iNO	*1.000*	*0.337*	
Driving pressure (cm H_2_0)			
Baseline	15 [14–17]	16 [14–17]	0.209
After iNO	14 [13–16]	13 [13–16]	1.000
*P* value before/after iNO	*0.221*	0.098	
RS compliance (ml cmH_2_0^−1^)			
Baseline	30.0 [21.8–36.7]	26.6 [20.2–31.8]	0.534
After iNO	33.9 [24.7–37.0]	30.0 [22.1–33.4]	0.407
*P* value before/after iNO	*0.345*	*0.073*	
ACP, *n* (%)	4 (33)	6 (27)	0.714
Prone positioning after iNO, *n* (%)	7 (58)	0 (0)	< 0.0001
CT scan features			
GGO	10 (83)	20 (91)	0.602
Consolidation	6 (50)	14 (64)	0.487
ICU mortality, *n* (%)	5 (42)	8 (36)	1.000

Change over time within groups was determined by Wilcoxon signed rank test (*P* value before/after iNO) and between groups were determined by Mann–Whitney test (*P* value)

*PEEP* positive end expiratory pressure, *ACP* acute cor pulmonale, *RS* respiratory system, *FiO*_*2*_ inspired fraction in oxygen, *GGO* ground glass opacities, *GGO* ground glass opacities, *CT scan* computerized tomography scanner, *ICU* intensive care unit

## Conclusion

If iNO improves PaO_2_/FiO_2_ ventilation/perfusion in the majority of COVID-19 patients with severe pneumonia, the causes of unresponsiveness to iNO remain unclear.

## Data Availability

Available on request.

## Abbreviation

iNO: Inhaled nitric oxide
